# Acquired angioedema and rituximab-induced acute thrombocytopenia in splenic marginal zone lymphoma

**DOI:** 10.2340/1651-226X.2025.43897

**Published:** 2025-07-09

**Authors:** Odd Terje Brustugun, Erik Waage Nielsen

**Affiliations:** a Section of Oncology, Drammen Hospital, Vestre Viken Hospital Trust, Drammen, Norway; b Institute of Clinical Medicine, University of Oslo, Oslo, Norway; c Department of Surgery, Nordland Hospital Trust, Bodø, Norway; d Institute of Clinical Medicine, University of Tromsø, Tromsø, Norway; e Faculty of Nursing and Health Sciences, Nord University, Bodø, Norway; f Department of Pain Medicine and Research, Oslo University Hospital and University of Oslo, Norway

**Keywords:** Lymphoma, angioedema, rituximab, thrombocytopenia, rituximab-induced acute thrombocytopenia

## Introduction and patient

A 75-year-old woman with a history of treated hypothyroidism (levothyroxine) and hypertension (losartan) presented with a sudden onset of severe tongue swelling upon waking. She described her tongue ‘as large as a meatball’. The swelling caused dyspnea, dysphagia, and a markedly hoarse voice, making communication nearly impossible. Emergency medical services were contacted and she was administered intramuscular 0.5 mg adrenaline with some subjective effect. Upon arrival at the hospital, she was given intramuscular 10 mg dexchlorpheniramine and intravenous 250 mg hydrocortisone and admitted for overnight observation. The swelling subsided significantly within a few hours.

She was advised to discontinue her angiotensin II receptor blocker (losartan), suspected of causing the angioedema.

In the days leading up to the episode, she found that her tongue felt rough to the touch. The evening prior, she reported feeling generally unwell and fatigued to a visiting friend. Over the preceding 2–3 years, she had intermittent episodes of diarrhea. No erythema marginatum or urticarial rash was observed before the episode. She had lost about 10% of her weight in the past few months.

She had no history of similar angioedema attacks, nor any other localized swelling episodes. There was no family history of angioedema. She had previously used estrogen in her 40s during suspected perimenopause without any associated symptoms.

**Figure 1:**
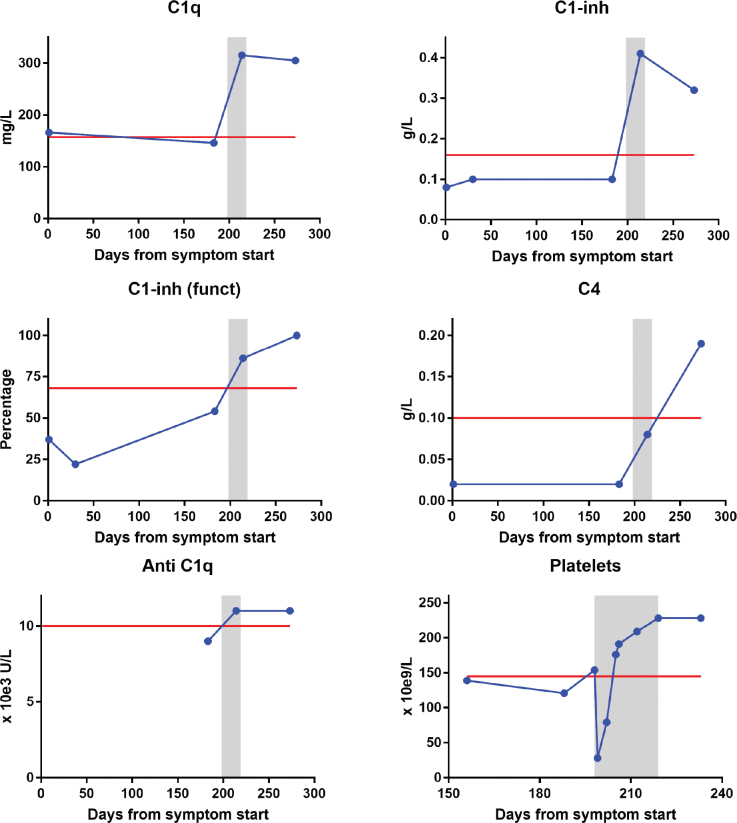
Levels of complement C1q (A), quantitative (B) and functional (C) C1-inhibitor, complement C4 (D) and autoantibodies directed against C1q (anti-C1q) (E) during the disease course. Platelet levels are shown before, during and after rituximab therapy (F). On x-axis, day 0 is the first day of angioedema symptoms. Red line is lower normal levels (except for anti C1q, where red line is upper normal level). Gray zone marks the period in which rituximab was given (4 weekly infusions).

Laboratory results revealed complement component C1q within the normal range, but both quantitative and functional C1-inhibitor deficiency, and low C4 levels (Figure 1A–D). A moderate lymphocytosis of 5.6 × 10e9 /L was noticed. Further radiological and hematological investigation confirmed the presence of a splenic marginal zone lymphoma, stage IVb with enlarged spleen (20 cm in craniocaudal diameter) and bone marrow affection (25% small B-cells). Serum protein electrophoresis was normal, thus no signs of monoclonal gammopathy of undetermined significance (MGUS).

Treatment with four weekly infusions of rituximab was decided and she was hospitalized one night after first two infusions due to risk of reappearance of angioedema. The morning after first rituximab infusion, a transient drop in platelet count to 28 × 10e9 /L was detected, from 154 × 10e9 /L the day before (Figure 1F). The platelet levels increased to 79 × 10e9 /L after 3 days, to normal level (176 × 10e9 /L) on day 6, and remained within normal limits during the next infusions. Following completion of rituximab therapy, the patient remained clinically stable, with no further angioedema episodes, improvement of fatigue and full resolution of previous gastrointestinal symptoms. CT scan 3 months later confirmed complete radiological response and she subsequently completed lymphoma treatment with four additional rituximab infusions with no complications.

Complement parameters and platelet counts returned to normal levels after rituximab therapy (Figure 1). Level of C1q that was initially within normal limits, rose markedly after rituximab infusion to what was likely its true baseline in the upper reference range.

## Discussion

This case fulfills clinical and laboratory criteria consistent with acquired angioedema (AAE) [1]. Apart from the moderately reduced C1q level, the complement profile of C1 inhibitor and C4 could be compatible with hereditary angioedema (HAE, type 1). However, several factors argue against this differential diagnosis: the patient experienced her first attack later in life, and the swelling was confined to the tongue and pharynx. Patients with HAE typically present in childhood or adolescence. This disease worsens dramatically by the use of estrogen containing pills and the swellings more often involve the lips, extremities, or gastrointestinal tract. These episodes are also frequently preceded by a reticular rash, erythema marginatum. The absence of this erythema in our patient and lack of swelling in other anatomical locations further argues against HAE. Bowel edema with diarrhea and colicky pain is characteristic of HAE, but it remains uncertain whether our patient’s episodes of diarrhea were bradykinin-mediated, although they resolved following normalization of C1 inhibitor levels. Our patient had no personal or family history of angioedema, tolerated estrogen therapy earlier in life, and presented with symptom onset at an older age.

Furthermore, her low C1q level prior to rituximab treatment supports the diagnosis of AAE as this finding is typically not seen in patients with HAE. C1q is a part of the pentamer complement 1 factor (C1). Reduced levels of C1q can point to a limited activation of the early complement cascade, as C3 is usually normal in AAE.

The co-occurrence of a lymphoma, along with normalization of C1-inhibitor and C4 values after rituximab therapy, supports a pathogenic link between lymphoproliferative disease and acquired complement consumption or inhibition [2, 3]. Approximately a third of patients with C1-inhibitor-related AAE are found with an underlying lymphoma, of which splenic marginal zone lymphoma seems to be most frequent [4].

The pathophysiological mechanism underlying the edema formation is shared between HAE and AAE: insufficient C1-inhibitor activity leads to uncontrolled kallikrein activation, with subsequent overproduction of bradykinin. Bradykinin acts via B2 receptors to induce vasodilation and increased vascular permeability, resulting in tissue edema [5].

Several mechanisms are proposed for the decreased C1-inhibitor levels in AAE: Increased consumption due to overactivation of the C1-complex alone [6], and/or by binding of an antibody to the antigen-recognition site (idiotype) of another antibody. This creates an immunocomplex that produces an excess of C1-activation and subsequent C1-inhibitor consumption [7].

Finally, patients may develop neutralizing antibodies directed against C1 inhibitor – either of the IgG or, more rarely, IgM subtype. These antibodies bind to functional domains of the C1-INH molecule and neutralize its protease-inhibitory activity, even if antigenic levels remain near normal. The result is uncontrolled activation of the classical complement pathway, excessive consumption of C1-INH, and increased generation of bradykinin, which leads to recurrent episodes of angioedema. Such neutralizing autoantibodies were not assessed in our patient due to short-term storage of routine samples and the absence of clinical suspicion of AAE at the time [8]. Re-evaluation of AAE cohorts has shown that such autoantibodies may coexist with lymphoma, and in some cases the monoclonal component itself corresponds to the anti–C1-inhibitor antibodies. These findings suggest that AAE may reflect a spectrum of B-cell proliferation, ranging from autoreactive clones to overt lymphoma [9]. MGUS is frequently identified in AAE and is considered part of this continuum [10] but was not seen in our patient. In 15% to 20% of AAE patients no hematological condition is found [11].

Plasma-derived C1-inhibitor was the first-line treatment for life-threatening attacks in AAE, although some patients become refractory due to neutralizing autoantibodies, in which case agents such as ecallantide or icatibant – proven effective in HAE – may offer a rational and favorable alternative [9].

Both AAE and systemic lupus erythematosus (SLE) can present with reduced C1q levels; however, in SLE this is typically accompanied by low C4 and C3 due to classical complement pathway activation, whereas in AAE only C1q and C4 are usually decreased, with C3 remaining normal. As above, SLE can in fact also cause angioedema in certain rare cases, in which case it is referred to as AAE [12].

Notably, our patient had low levels of anti-complement component 1q antibodies (aC1q) further supporting the distinction from SLE (Figure 1E) [13]. Anti-C1q antibodies are detected in up to approximately 50% of patients with SLE and appear to be even more prevalent among those with lupus nephritis [14]. Laboratory characteristics in angioedema subtypes are presented in Table 1.

**Table 1:** Laboratory characteristics in angioedema subtypes.

Disease	C1-INH Ag	C1-INH Fc	C4	C2	C3	C1q	aC1q	Genetic	MGUS	C1-INH Ab
AAE	↓ (N)	↓ (N)	↓ (N)	↓ (N)	N	↓ (N)	N	–	+ / -	+ / -
HAE type 1	↓	↓	↓	↓	N	N	N	Yes	–	–
HAE type 2	N /↑	↓	↓	↓	N	N	N	Yes	–	–
SLE	N	N	↓	↓	↓	↓	↑	–	–	–
HAE nC1-INH	N	N	N	N	N	N	N	Yes	–	–
ACE-INH ANGIOEDEMA	N	N	N	N	N	N	N	(?)	–	–

AAE: acquired angioedema; HAE type 1: hereditary angioedema with low C1 inhibitor (C1-INH) levels; HAE type 2: hereditary angioedema with normal or elevated C1-INH levels but reduced function; SLE: systemic lupus erythematosus; HAE nC1-INH: hereditary angioedema with normal C1 inhibitor (formerly referred to as HAE type 3); ACE-INH angioedema: angioedema induced by angiotensin-converting enzyme inhibitors. C1-INH Ag: C1 inhibitor antigenic level; C1-INH Fc: C1 inhibitor functional activity; C4: complement component 4; C2: complement component 2; C3: complement component 3; C1q: complement component 1q; aC1q: anti-complement component 1q antibodies; MGUS: monoclonal gammopathy of undetermined significance; C1-INH Ab: autoantibodies against C1 inhibitor.

Although losartan, an angiotensin II receptor blocker could have acted as a trigger for the angioedema episode in a predisposed individual, as described for ACE-inhibitors by Kleiner et al. [15], and angioedema due to angiotensin II receptor blockers typically affects the tongue, it is extremely rare. Only a few cases of angiotensin II receptor blocker-induced AAE have been reported, it is angiotensin converting enzyme inhibitors that account for nearly all instances [16]. In contrast, our patient exhibited biochemical features consistent with acquired C1-inhibitor deficiency, including a concurrently low C1q level. The likelihood of two unrelated and extremely rare mechanisms – losartan-induced angioedema and lymphoma-associated C1-INH deficiency – co-occurring in the same patient is markedly lower than that of a single, unified explanation: B-cell lymphoma with complement-mediated angioedema due to acquired C1 inhibitor deficiency.

In our patient with splenic marginal zone lymphoma, a significant drop in platelet count was observed following rituximab administration, with spontaneous recovery within hours. This phenomenon, known as rituximab-induced acute thrombocytopenia (RIAT), has been documented in the literature [17], particularly among patients with B-cell lymphomas such as splenic marginal zone lymphoma [18]. The precise mechanism remains unclear; however, proposed explanations include immune-mediated platelet destruction via complement activation, formation of immune complexes, or interactions involving Fc receptors on platelets. Importantly, the rapid onset and resolution of thrombocytopenia suggests a peripheral destruction mechanism rather than impaired platelet production. Clinicians should be aware of this rare but potentially serious adverse event, and routine monitoring of platelet counts following rituximab infusion is advisable, especially in patients with underlying lymphoproliferative disorders.

Bradykinin-mediated angioedema, as seen in both hereditary and acquired forms, may occur unpredictably and can recur after prolonged asymptomatic intervals, as observed in our patient. In this context, the diagnosis of AAE remains valid, as C1-inhibitor levels remained persistently low throughout the entire period.

To our knowledge, this is the first reported case demonstrating coexisting AAE and RIAT in the context of B-cell lymphoma. While both conditions are individually linked to B-cell lymphoproliferative disorders and rituximab, their co-occurrence has not been described. The rapid, transient platelet drop suggests a peripheral immune-mediated mechanism, possibly involving complement activation or Fc receptor interactions.

This case underscores the need to consider AAE in older patients with unexplained swelling, and to actively investigate for an underlying lymphoproliferative malignancy when such a diagnosis has not yet been established.

## Data Availability

The clinical data in this article are retained in the individual’s medical journal, hence not available in public domain.
